# 1539. A Nine-Gene Blood-Based Signature Meets the World Health Organization Target Product Profiles for Diagnosis of Active Tuberculosis and Predicting Progression from Latent to Active Disease

**DOI:** 10.1093/ofid/ofac492.094

**Published:** 2022-12-15

**Authors:** Sanjana Gupta, Aditya Rao, Madeleine Scott, Valeriu Crudu, Timothy Rodwell, Donald Catanzaro, Antonino Catanzaro, Purvesh Khatri

**Affiliations:** Stanford University, SUNNYVALE, CA; Stanford University, SUNNYVALE, CA; Stanford University, SUNNYVALE, CA; Nicolae Testemitanu State University of Medicine and Pharmacy, Chisinau, Chisinau, Moldova; University of California, San Diego, La Jolla, California; University of Arkansas, Fayetteville, Arkansas; University of California, San Diego, La Jolla, California; Stanford University, SUNNYVALE, CA

## Abstract

**Background:**

As part of its End TB strategy, the WHO has identified the need for non-sputum-based diagnostics that meet target product profiles (TPP) of 90% sensitivity and 70% specificity for diagnosis of Active TB (ATB) and 75% sensitivity and specificity for predicting progression from Latent TB (LTB) to ATB. The successful translation of a 3-gene blood-based signature, identified using diverse datasets, into a prototype point-of-care diagnostic, that meets the WHO TPPs, has demonstrated the power of integrating large amounts of heterogeneous data to identify generalizable disease signatures. We hypothesized that integration of more diverse datasets, comprising patients with ATB or other inflammatory lung diseases, would identify novel, robust signatures, for diagnosing ATB and predicting progression from LTB to ATB, that meet the WHO TPPs.

**Methods:**

Using multi-cohort analyses, we integrated and analyzed data from 3,615 peripheral blood samples, in 49 publicly available transcriptomic datasets (discovery cohorts), from healthy controls and patients with LTB, ATB, and other diseases (COPD, viral infections, sarcoidosis, etc.). We used data from (1) 3,836 blood samples in 28 retrospective datasets and (2) 360 prospectively collected blood samples, from a household contact study in Moldova, as validation cohorts.

**Results:**

Using the discovery cohorts we identified a 9-gene signature for diagnosing ATB patients from healthy controls, or individuals with LTB or other diseases. The signature achieved 90% sensitivity and 82% specificity in retrospective validation (Figure 1A) and 90% sensitivity and 69% specificity in the prospective cohort from Moldova (Figure 1B). In a longitudinal cohort of adolescents, the 9-gene signature predicted progression from LTB to ATB up to 1 year prior to sputum conversion with 76% sensitivity and 83% specificity (Figure 1C). Finally, the signature predicted prolonged lung inflammation post-treatment in the Catalysis Treatment Response Cohort (Figure 1D).

9-gene TB signature validates in independent retrospective and prospective cohorts.

(A) ROC curves for comparing ATB vs. all other samples (All), or individually comparing ATB vs. Healthy, LTBI or Other Disease (OD) samples in independent retrospective validation and (B) a prospective Moldova cohort. (C) ROC curves comparing progressor and non-progressor samples collected at different time points in the Adolescent Cohort Study (ACS), for the 9-gene signature in solid lines and a previous 3-gene signature in dashed lines, (D) Distribution of the 9-gene score at different time points post-treatment in the Catalysis Treatment Response Cohort Study, for individuals with persistent lung inflammation at 24 weeks compared with those who had clear lungs at 24 weeks.

**Conclusion:**

Overall, the 9-gene signature meets the WHO TPPs required for the End TB strategy.

**Disclosures:**

**Purvesh Khatri, PhD**, Cepheid, Inc.: Advisor/Consultant|Inflammatix, Inc.: Advisor/Consultant|Inflammatix, Inc.: Inflammatix is in negotiations to license the 9-gene signature discussed here.|Inflammatix, Inc.: Stocks/Bonds.

